# Supplementary material for: SETD6-mediated methylation of PPARγ establishes a transcriptional feedback circuit promoting lipid accumulation in liver-derived cells
Source: eLife. 2026 Aug 28;15:RP111542. doi: 10.7554/eLife.111542 (PMC13524377; doi:10.7554/eLife.111542)
Supplement: Peer review history

# Editor report: eLife Assessment

**DOI:** 10.7554/eLife.111542.3.sa0

**Authors:** Xiaobing Shi

**Affiliations:** Van Andel Institute United States

**Keywords:** Convincing, Important

This manuscript presents **important** new findings showing that the transcription factor and regulator of lipid and glucose metabolism PPARγ is methylated by the enzyme SETD6. The data **convincingly** demonstrate that methylation of PPARγ by SETD6 regulates its function in controlling transcription of lipid storage and metabolism genes and thereby modulates lipid accumulation in liver cells. This work uncovers a new role for post-translational modulation by lysine methylation in controlling transcription factor activity and lipid metabolism in a relevant physiological context.

# Referee report: Reviewer #1 (Public review)

**DOI:** 10.7554/eLife.111542.3.sa1

Summary:

In this manuscript from the Levy lab, the authors investigate whether SETD6 regulates hepatic lipid accumulation through direct methylation of PPARγ. They show that SETD6 binds and mono-methylates PPARγ at K170 and provide evidence that this modification enhances PPARγ occupancy at target promoters, promotes expression of lipid metabolism genes, as well as facilitates lipid droplet accumulation in HepG2 cells. The authors also find a positive feedback loop or circuit in which PPARγ activates SETD6 transcription in a methylation-dependent manner, thereby reinforcing this lipogenic program. Overall, the work presents a novel SETD6-PPARγ regulatory axis linking lysine methylation to transcriptional control of lipid storage genes, with possible relevance to NAFLD-associated biology.

In all, I find this to be an important paper that describes and advances a new regulatory pathway that has significance to human health and disease. It would also be of interest to a broad audience. That said, there are also some concerns that the authors should address, as outlined below.

Major concerns (pertains to rigor - highest priority)

(1) Overall, the work presented is of high quality and the data nicely support the conclusions; however, a few panels should be strengthened that have missing controls or information:

a. The co-IP panel in Fig. 1B lacks a lane where HA SETD6 is expressed without PPARγ. This control is needed to verify that the SEDT6-HA signal depends on PPARγ.

b. In Fig. 1C, the authors should show that the co-IP works in both directions (include IP for PPARγ/blot for SETD6). I am a bit confused also over the labeling with IP on the left and on top of the panel next to the beads label. More importantly, the data would be stronger if the authors take advantage of a deletion line to validate the co-IP is specific to the presence of both.

c. The same IP labeling issue exists for Fig 3B (label is on the same and on top).

d. Antibody information (e.g., where the pan-methyl Ab comes from and at what dilutions they are used at) is missing.

Nice to have experiments (medium priority - strongly consider)

(2) A missing gap is how K170me1 contributes to DNA binding and gene transcription. One possibility is that methylation enhances the DNA binding activity of PPARγ. Given the authors have all of the reagents, it would be possible to perform a gel shift assay (or other approach) with and without SETD6-mediaetd methylation. Is DNA binding affected/enhanced?

(3) Along these lines, I wonder if there is another possibility: could SETD6-mediated methylation of PPARγ drive SETD6-PPARγ interaction? In other words, in the K170R, is SETD6 still even associated with PPARγ, and this interaction is required for promoter recruitment? Alternatively, would a catalytic dead version of SETD6 fail to associate with PPARγ? Currently, no experiments test the impact of an unmethylatable version of PPARγ or catalytic dead version of SETD6 on SETD6-PPARγ interaction or SETD6 recruitment to promoters.

Minor concerns (text and figure display)

(4) The text has multiple typos and grammatical errors.

Comments on revised version.

Great job on addressing the comments. It is a nice study.

# Referee report: Reviewer #2 (Public review)

**DOI:** 10.7554/eLife.111542.3.sa2

Summary:

In this work, the authors investigated the regulation of the transcription factor PPARγ by the post-translational modification lysine methylation The data demonstrate that the lysine methyltransferase SETD6 targets PPARγ for methylation using biochemical and cell-based assays. Methylation of PPARγ occurs in its DNA binding domain, and the authors demonstrate that loss of methylation limits PPARγ chromatin binding, particularly to lipid storage and metabolism genes promoters. As a physiological output, the authors demonstrate that deletion of SETD6 and loss of PPARγ methylation also disrupt lipid droplet accumulation in hepatocytes. In addition, the authors uncover a positive feedback loop in which SETD6 methylation of PPARγ also regulates its binding to the SETD6 promoter and expression of the gene.

Strengths:

One of the key strengths of this manuscript is the novelty of the findings in terms of identifying a new mode of regulation of PPARγ that modulates its chromatin association in cells and thereby regulating lipid metabolism genes. The authors nicely combine biochemical studies of SETD6 activity with cell-based assays investigating PPARγ and SETD6 function in regulating lipid storage. Data supporting this conclusion is largely convincing and frequently, multiple assays are used to provide sufficient support to the conclusions. This work therefore expands regulatory modes of PPARγ and identifies a new target for SETD6, an enzyme that targets a number of other transcription factors. Furthermore, the regulatory loop that controls SETD6 expression via PPARγ methylation is likely important for understanding SETD6 function in different cell types that have high levels of lipid accumulation or regulation. The gene expression and lipid accumulation assays are useful for testing the physiological outcome of loss of SETD6 activity or PPARγ methylation directly. In the revised manuscript, the authors have added useful structural modeling to better define potential roles of methylation of PPARγ in regulating its function, particularly relative to DNA binding, and to better define the physical interaction between PPARγ and SETD6.

Weaknesses:

The revised manuscript substantially improved on the presentation of the data and broadened the discussion to provide more context to both the role of SETD6 and to elaborate on potential mechanisms by which methylation impacts PPARγ function and under what physiological conditions this interaction and regulation is important. This improves and strengthens the manuscript and its impact overall.

Comments on revised version.

The authors addressed all of my major concerns following this round of review and I do not have additional recommendations. The presentation of the manuscript including text and figures is improved compared to the previous version. I have updated my public review to reflect these changes.

# Author comment: Author response

**DOI:** 10.7554/eLife.111542.3.sa3

**Authors:** Noa Nashnaz, Dana Goldberg, Maayan Abramov, Anand Chopra, Tamar Rosiecki, Habib Muallem, Yulia Haim, Tzofit Elbaz Biton, Liron Levin, Raz Zarivach, Michal Feldman, Assaf Rudich, Dan Levy

**Affiliations:** Ben-Gurion University of the Negev beer sheva Israel; Ben-Gurion University of the Negev beer sheva Israel; Ben-Gurion University of the Negev beer sheva Israel; Ben-Gurion University of the Negev beer sheva Israel; Ben-Gurion University of the Negev beer sheva Israel; Ben-Gurion University of the Negev beer sheva Israel; Ben-Gurion University of the Negev beer sheva Israel; Ben-Gurion University of the Negev beer sheva Israel; Ben-Gurion University of the Negev beer sheva Israel; Ben-Gurion University of the Negev beer sheva Israel; Ben-Gurion University of the Negev beer sheva Israel; Ben-Gurion University of the Negev beer sheva Israel; Ben-Gurion University of the Negev beer sheva Israel

The following is the authors’ response to the original reviews.

**Public Reviews:**

**Reviewer #1 (Public review):**

Summary:

In this manuscript from the Levy lab, the authors investigate whether SETD6 regulates hepatic lipid accumulation through direct methylation of PPARγ. They show that SETD6 binds and monomethylates PPARγ at K170, and provide evidence that this modification enhances PPARγ occupancy at target promoters, promotes expression of lipid metabolism genes, as well as facilitates lipid droplet accumulation in HepG2 cells. The authors also find a positive feedback loop or circuit in which PPARγ activates SETD6 transcription in a methylation-dependent manner, thereby reinforcing this lipogenic program. Overall, the work presents a novel SETD6PPARγ regulatory axis linking lysine methylation to transcriptional control of lipid storage genes, with possible relevance to NAFLD-associated biology.

In all, I find this to be an important paper that describes and advances a new regulatory pathway that has significance to human health and disease. It would also be of interest to a broad audience. That said, there are also some concerns that the authors should address, as outlined below.

We are grateful to the reviewer for the positive feedback and appreciation of our work.

Major concerns (pertains to rigor - highest priority)

(1) Overall, the work presented is of high quality, and the data nicely support the conclusions; however, a few panels should be strengthened that have missing controls or information:

(a) The co-IP panel in Figure 1B lacks a lane where HA SETD6 is expressed without PPARγ. This control is needed to verify that the SEDT6-HA signal depends on PPARγ.

We thank the reviewer for this valuable suggestion. The overexpression co-immunoprecipitation experiment referred to by the reviewer has been moved to Supplementary Figure S1 in the revised manuscript. In this experiment, immunoprecipitation was performed using an anti-FLAG antibody to pull down FLAG-tagged PPARγ. In the absence of FLAG-PPARγ, the anti-FLAG immunoprecipitation does not recover a bait protein, and therefore HA-SETD6 is not expected to be specifically immunoprecipitated. Thus, an HA-SETD6-only condition would primarily serve as a negative control for the anti-FLAG pull-down rather than provide additional information regarding the specificity of the interaction.

Importantly, in the revised manuscript we have substantially strengthened the evidence supporting the SETD6–PPARγ interaction by adding two independent complementary experiments. First, we included a reciprocal endogenous co-immunoprecipitation (new Figure 2B), demonstrating that endogenous PPARγ co-immunoprecipitates with endogenous SETD6. Second, we added an independent proximity ligation assay (PLA) (new Figure 2D), which further confirms the interaction between SETD6 and PPARγ in cells. Together with the in vitro binding assay presented in Figure 2A, these orthogonal approaches provide compelling evidence for the specificity of the SETD6–PPARγ interaction. Therefore, we believe that the requested HA-SETD6-only control would not provide additional mechanistic insight beyond the comprehensive validation now included in the revised manuscript.

(b) In Figure 1C, the authors should show that the co-IP works in both directions (include IP for PPARγ/blot for SETD6). I am a bit confused also over the labeling with IP on the left and on top of the panel next to the beads label. More importantly, the data would be stronger if the authors took advantage of a deletion line to validate that the co-IP is specific to the presence of both.

We thank the reviewer for this helpful suggestion. We have revised the manuscript to strengthen the evidence supporting the endogenous interaction between SETD6 and PPARγ. Specifically, we now include a reciprocal endogenous co-immunoprecipitation (new Figure 2B), demonstrating that endogenous SETD6 co-immunoprecipitates with endogenous PPARγ and, conversely, that endogenous PPARγ co-immunoprecipitates with endogenous SETD6. These reciprocal experiments independently validate the specificity of the interaction.

In addition, we have revised the figure layout and labeling to more clearly distinguish the immunoprecipitating antibody from the bead control, thereby addressing the reviewer's concern regarding the presentation of the co-immunoprecipitation data.

Although we did not perform the co-immunoprecipitation in a depletion/knockout background, we believe that the combination of reciprocal endogenous co-immunoprecipitation (New Figure 2B), the independent proximity ligation assay (New Figure 2D), and the direct in vitro binding assay (Figure 2A) provides multiple orthogonal lines of evidence supporting a specific interaction between SETD6 and PPARγ.

(c) The same IP labeling issue exists for Figure 3B (label is on the same and on top).

We have revised the labeling in Figure 3B to clearly distinguish the immunoprecipitating antibody from the bead control, thereby improving the clarity of the figure.

(d) Antibody information (e.g., where the pan-methyl Ab comes from and at what dilutions they are used at) is missing.

We thank the reviewer for pointing this out. We have now added the missing information regarding the pan-methyl antibody to the Materials and Methods section, including the supplier, catalogue number, and experimental conditions used. Specifically, the pan-methyl antibody used in this study was purchased from Abcam (ab23366) and was used at a 1:500 dilution for western blot analysis and 2 μg per reaction for immunoprecipitation experiments.

Nice to have experiments (medium priority - strongly consider)

(2) A missing gap is how K170me1 contributes to DNA binding and gene transcription. One possibility is that methylation enhances the DNA-binding activity of PPARγ. Given that the authors have all of the reagents, it would be possible to perform a gel shift assay (or other approach) with and without SETD6-mediated methylation. Is DNA binding affected/enhanced?

We thank the reviewer for raising this important point. To investigate whether K170 methylation could directly affect PPARγ binding to DNA, we performed structural modeling based on the available co-crystal structure of PPARγ bound to DNA (PDB: 3DZU). As shown in the new Figure 6F, K170 is positioned near the DNA-binding region; however, the modeled K170me1 side chain is predicted to face away from the DNA interface and does not appear to sterically interfere with the PPARγ–DNA interaction. In addition, modeling of multiple K170me1 rotamers did not suggest any major disruption of the DNA-bound conformation.

These observations suggest that K170 methylation is unlikely to directly alter the intrinsic DNA-binding affinity of PPARγ. In contrast, our ChIP-qPCR experiments demonstrate that K170 methylation positively regulates PPARγ occupancy at target promoters in cells. Together, these findings support a model in which K170 methylation promotes PPARγ chromatin association and transcriptional activity through mechanisms other than direct modulation of DNA binding, such as altered cofactor recruitment or protein–protein interactions.

We agree with the reviewer that future biochemical approaches, including EMSA/gel shift assays or quantitative DNA-binding measurements, will be valuable to directly determine whether K170 methylation affects the intrinsic DNA-binding affinity of PPARγ. We have incorporated this new structural analysis and the corresponding discussion into the revised manuscript.

(3) Along these lines, I wonder if there is another possibility: could SETD6-mediated methylation of PPARγ drive SETD6-PPARγ interaction? In other words, in the K170R, is SETD6 still even associated with PPARγ, and this interaction is required for promoter recruitment? Alternatively, would a catalytic dead version of SETD6 fail to associate with PPARγ? Currently, no experiments test the impact of an unmethylatable version of PPARγ or a catalytic dead version of SETD6 on SETD6-PPARγ interaction or SETD6 recruitment to promoters.

We thank the reviewer for this insightful suggestion. To address whether SETD6 catalytic activity is required for its association with PPARγ, we performed an additional PLA experiment comparing SETD6 WT and the catalytic mutant SETD6 Y285A. As shown in the revised New Figure 2D, both SETD6 WT and SETD6 Y285A showed comparable proximity to PPARγ in cells, indicating that SETD6 catalytic activity is not required for the physical association between SETD6 and PPARγ.

These findings support a model in which SETD6 first recognizes and binds PPARγ independently of its catalytic activity. Subsequent methylation of PPARγ at K170 is therefore likely to regulate the downstream functional consequences of this interaction, including enhanced chromatin occupancy and transcriptional activation, rather than the initial SETD6–PPARγ association itself.

In addition, we generated using AlphaFold a structural model of the SETD6–PPARγ complex as a supportive visualization (New figure S2). Given the limited confidence of the prediction, we interpret this model cautiously and include it in the Supplementary Information rather than the main figures. We agree with the reviewer that future studies examining SETD6 recruitment to PPARγ target promoters and the effect of the PPARγ K170R mutant on SETD6–PPARγ association will further refine the molecular mechanism.

Minor concerns (text and figure display)

(4) The text has multiple typos and grammatical errors, and there are some issues with the figure display.

We thank the reviewer for this comment. We carefully revised the manuscript to correct typographical and grammatical errors throughout the text and also addressed the figure display issues noted by the reviewer.

**Reviewer #2 (Public review):**

Summary:

In this work, the authors investigated the regulation of the transcription factor PPARγ by the post-translational modification lysine methylation. The data demonstrate that the lysine methyltransferase SETD6 targets PPARγ for methylation using biochemical and cell-based assays. Methylation of PPARγ occurs in its DNA binding domain, and the authors demonstrate that loss of methylation limits PPARγ chromatin binding, particularly to lipid storage and metabolism gene promoters. As a physiological output, the authors demonstrate that deletion of SETD6 and loss of PPARγ methylation also disrupt lipid droplet accumulation in hepatocytes. In addition, the authors uncover a positive feedback loop in which SETD6 methylation of PPARγ also regulates its binding to the SETD6 promoter and expression of the gene.

Strengths:

One of the key strengths of this manuscript is the novelty of the findings in terms of identifying a new mode of regulation of PPARγ that modulates its chromatin association in cells and thereby regulates lipid metabolism genes. The authors nicely combine biochemical studies of SETD6 activity with cell-based assays investigating PPARγ and SETD6 function in regulating lipid storage. Data supporting this conclusion is largely convincing, and frequently, multiple assays are used to provide sufficient support to the conclusions. This work therefore expands regulatory modes of PPARγ and identifies a new target for SETD6, an enzyme that targets a number of other transcription factors. Furthermore, the regulatory loop that controls SETD6 expression via PPARγ methylation is likely important for understanding SETD6 function in different cell types that have high levels of lipid accumulation or regulation. The gene expression and lipid accumulation assays are useful for testing the physiological outcome of loss of SETD6 activity or PPARγ methylation directly.

We thank the reviewer for his/her positive feedback on the manuscript.

Weaknesses:

The data presented in the manuscript are largely convincing in support of the authors' conclusions; however, there are some errors in the presentation of the figures and some issues in the text that would benefit from editing. Furthermore, there are some important questions not fully addressed in the results or discussion.

It would be great if the authors could speculate more on the diverse roles of SETD6 in methylated transcription factors and/or provide more context regarding the conditions that are likely to support methylation of PPARγ by SETD6.

We thank the reviewer for this important suggestion. In the revised Discussion, we expanded the manuscript to better place our findings within the broader context of SETD6-mediated regulation of transcription factors. Previous studies from our group and others demonstrated that SETD6 methylates multiple chromatin-associated transcriptional regulators, including RelA, E2F1, TWIST1, and BRD4, thereby modulating transcriptional selectivity, chromatin occupancy, and cofactor recruitment. We now discuss the possibility that SETD6 functions as a context-dependent signalling integrator that selectively regulates transcription factor activity through lysine methylation under distinct physiological conditions.

In addition, we expanded the Discussion regarding potential cellular contexts that may favour PPARγ methylation by SETD6. Because PPARγ activity is strongly induced during lipid overload and fatty acid exposure, conditions associated with steatosis and metabolic stress may enhance the functional importance of SETD6-dependent methylation. We also discuss the possibility that chromatin accessibility, ligand-dependent activation of PPARγ, and metabolic signaling pathways may collectively influence the formation and stability of the SETD6–PPARγ complex.

Also, while a potential cross-talk between methylation and phosphorylation is described in the discussion, it would be great to provide more structural insight into how this might regulate DNA binding of PPARγ and/or discuss whether there are other possibilities given the location of the target lysine in the DNA binding domain.

We thank the reviewer for this valuable suggestion. To provide additional structural insight into the potential interplay between methylation and phosphorylation within the PPARγ DNA-binding domain, we performed structural modeling based on the published PPARγ–DNA co-crystal structure (PDB: 3DZU). As shown in the new Figure S6, the modeled K170me1 side chain is predicted to face away from the DNA interface and does not introduce steric clashes with DNA, suggesting that K170 methylation is unlikely to directly alter the DNA-binding interface through steric effects. In contrast, phosphorylation of the neighboring residue T166 is predicted to introduce multiple intramolecular steric clashes within the DNA-binding domain. These structural changes could influence the local conformation or dynamics of the DNA-binding domain and thereby indirectly modulate PPARγ DNA binding or transcriptional activity.

In addition, as suggested by the reviewer, we expanded the Discussion to consider alternative mechanisms by which K170 methylation may regulate PPARγ function. While our ChIP-qPCR experiments demonstrate that K170 methylation positively regulates PPARγ chromatin occupancy at target promoters, the structural modeling suggests that this effect is unlikely to arise from direct steric modulation of the DNA interface. Instead, K170 methylation may influence chromatin occupancy by regulating protein–protein interactions, cofactor recruitment, local conformational dynamics, or other chromatin-associated mechanisms. We have incorporated these new structural analyses and the expanded discussion into the revised manuscript.

**Recommendations for the authors:**

**Reviewer #1 (Recommendations for the authors):**

(1) The KEGG panels are too low resolution to read.

We thank the reviewer for this comment. We improved the resolution of the KEGG pathway enrichment panels and, as suggested by the reviewer (see below), we separated the upregulated and downregulated gene sets to improve clarity and readability. These changes are now reflected in the revised new Figures 5C, 5D, 6B and 6C.

(2) Figure 3 panel D is hard to visualize; a better version or repeat experiment is needed.

We thank the reviewer for this comment. To address this concern, we replaced the original Figure 3D with a new independent experiment that more clearly demonstrates the methylation of endogenous PPARγ by SETD6. We believe that the new data provide substantially stronger evidence and improve the clarity of the revised manuscript.

(3) There is a typo in Figure 2A (line present in "S" of Signal).

We thank the reviewer for pointing out this typo. The error in Figure 2A has now been corrected in the revised manuscript.

**Reviewer #2 (Recommendations for the authors):**

Overall, the experiments and analyses presented are sufficient to support the conclusions and interpretations of the work. However, there are some issues of presentation and writing that are worth addressing. These comments are listed below:

(1) My only substantial recommendation is to improve the discussion to provide more context to the findings in terms of both the larger role of SETD6 in methylating transcription factors (some of whom also regulate its expression) and the potential modes through which PPARγ DNA binding activity could be regulated by methylation.

We thank the reviewer for this insightful suggestion. In the revised manuscript, we substantially expanded the Discussion to better place our findings within the broader context of SETD6mediated regulation of transcription factors. We now discuss previous studies demonstrating that SETD6 methylates multiple chromatin-associated transcriptional regulators, including RelA, E2F1, TWIST1, and BRD4, thereby modulating chromatin occupancy, cofactor recruitment, and transcriptional selectivity. We further propose that SETD6 functions as a context-dependent signaling regulator that integrates distinct cellular pathways through the selective lysine methylation of transcription factors.

In addition, we expanded the Discussion regarding the potential mechanisms by which PPARγ K170 methylation regulates transcriptional activity. We incorporated new structural modeling based on the published PPARγ–DNA co-crystal structure (PDB: 3DZU), which suggests that K170 methylation is unlikely to directly alter the DNA-binding interface through steric effects, whereas phosphorylation of the neighboring residue T166 may induce intramolecular steric clashes within the DNA-binding domain. We also expanded the Discussion to consider alternative mechanisms by which K170 methylation may regulate PPARγ function, including modulation of chromatin occupancy, local conformational dynamics, protein–protein interactions, and recruitment of transcriptional cofactors or chromatin-associated proteins. Finally, we discuss that future biochemical studies will be important to determine whether K170 methylation also influences the intrinsic DNA-binding affinity of PPARγ.

Can the authors incorporate any other published structural data to speculate on the role of methylation or describe more about how it is expected that the methylation-phosphorylation crosstalk modulates DNA binding? This type of discussion would better highlight the potential importance of this new modification on PPARγ.

We thank the reviewer for this important suggestion. In the revised manuscript, we expanded the Discussion and incorporated additional structural analyses (new Figure 6F and new figure S6) based on the published PPARγ–DNA co-crystal structure (PDB: 3DZU). K170 is positioned within the DNA-binding domain, between the two zinc-finger motifs that mediate DNA recognition and stabilization on PPRE-containing DNA. Our structural modeling predicts that the K170me1 side chain is oriented away from the DNA interface and does not introduce steric clashes with DNA, suggesting that methylation is unlikely to directly alter the DNA-binding interface through steric effects. Nevertheless, lysine methylation can influence protein surface properties, protein– protein interactions, and recognition by regulatory binding partners, raising the possibility that K170 methylation modulates PPARγ chromatin occupancy or promoter selectivity through indirect mechanisms.

In contrast, structural modeling predicts that phosphorylation of the neighboring residue T166 introduces multiple intramolecular steric clashes within the DNA-binding domain. These clashes could alter the local conformation or dynamics of the DNA-binding domain and thereby indirectly influence PPARγ–DNA interactions and transcriptional activity. Together, these observations suggest that K170 methylation and T166 phosphorylation may represent a regulatory crosstalk that fine-tunes PPARγ function through distinct structural mechanisms.

We further expanded the Discussion to consider additional, non-mutually exclusive mechanisms by which K170 methylation may regulate PPARγ function, including modulation of chromatin occupancy, protein–protein interactions, cofactor recruitment, and stabilization of transcriptional complexes at target genes. While these possibilities require further mechanistic investigation, we agree with the reviewer that these structural considerations highlight the potential regulatory importance of this newly identified PPARγ modification.

(2) In the gene expression experiments presented in Figure 5, it would be useful if the GO terms were described as enriched in either the up- or down-regulated gene sets. From the way it is presented, it is not clear if specific categories of genes are found enriched in those upregulated or downregulated upon KO of SETD6. This is also true for the experiments presented in Figure 6 regarding the PPARγ mutant.

We thank the reviewer for this important suggestion. In the revised manuscript, we separated the pathway enrichment analyses into upregulated and downregulated gene sets for both the SETD6 knockout RNA-sequencing experiments (Figure 5) and the PPARγ WT versus K170R mutant analysis (Figure 6). This revision provides improved clarity regarding which biological pathways are positively or negatively associated with SETD6 depletion or disruption of PPARγ K170 methylation. The updated KEGG enrichment analyses are now presented in the revised new Figures 5C, 5D, 6B and 6C.

In addition, if there is a significant overlap in genes misregulated in both mutants, this could be shown in the figure.

We thank the reviewer for this suggestion. We compared the differentially expressed genes identified in the SETD6 KO cells and the PPARγ K170R mutant cells to evaluate the extent of overlap between the two datasets. However, we did not observe a substantial or statistically significant overlap in misregulated genes under the thresholds used in our analysis. Therefore, we decided not to include this comparison in the revised figure. Nevertheless, both datasets consistently showed enrichment for pathways associated with lipid metabolism and PPAR signaling, supporting a functional connection between SETD6 and PPARγ-mediated transcriptional regulation.

(3) There are some typos and grammatical errors throughout the work, and it should be carefully edited. One example is the following heading: PPARγ K170 methylation by SETD6 regulates mediates lipid droplets formation.

We thank the reviewer for this comment. The manuscript was carefully revised to correct typographical and grammatical errors throughout the text. In particular, the heading mentioned by the reviewer was corrected in the revised manuscript.

Minor errors in the figures:

(1) Formatting issue in the labeling for the x-axis of Figure 1E.

We thank the reviewer for pointing out this formatting issue. The labeling of the x-axis in Figure 1E has been corrected in the revised manuscript.

(2) Figure 2B - HA-SETD6 should be labeled as minus for the first lane.

We thank the reviewer for pointing this out. We corrected the labeling in Figure 2B (now Figure S1), and the first lane is now properly indicated as negative for HA-SETD6.

(3) Figure 2D - Labeling needs improvement. Is this FLAG-PPARγ? "NC" was not defined in the legend. If negative control, what type?

We thank the reviewer for this comment. We improved the labeling and figure legend of Figure 2D for clarity. Specifically, we now clearly indicate that the experiment was performed using Flag-PPARγ, and we defined “NC” in the legend as the negative control condition. In addition, during the revision process we noticed that the original PLA experiment was performed in HeLa cells rather than HepG2 cells, as previously indicated. This has now been corrected throughout the revised manuscript.

(4) Figure 3 - The CRSIPR control should be described somewhere in the legend or the methods.

We thank the reviewer for this comment. We clarified the description of the CRISPR control cells in the Materials and Methods section. Specifically, we now explicitly state that the CRISPR control (CT) cells were generated using the empty lentiCRISPR vector without SETD6-targeting sgRNAs.

(5) Figure 5B and 6A - The legend is not labeled nor defined in the text- fold-change, log2 fold change, z score?

We thank the reviewer for pointing this out. We revised the figure legends for Figures 5B and 6A to explicitly define the heatmap scale and normalization method. Specifically, we now indicate that the heatmaps represent normalized gene expression values displayed as Z-scores.
